# Wholesale funding and liquidity creation

**DOI:** 10.1007/s11156-022-01081-9

**Published:** 2022-07-05

**Authors:** George Kladakis, Lei Chen, Sotirios K. Bellos

**Affiliations:** 1 The Business School, Edinburgh Napier University, 219 Colinton Rd, EH14 1DJ Edinburgh, UK; 2 Loughborough University, Loughborough, UK; 3 South East European Research Centre (SEERC), CITY College, University of York Europe Campus, Thessaloniki, Greece

**Keywords:** Wholesale funding, Liquidity creation, Bank liquidity, Lending, Moral hazard, G20, G21, G32

## Abstract

We examine the relationship between wholesale funding and liquidity creation using a sample of 825 banks in 84 countries during the post-crisis period of 2010–2020. We find that asset-side liquidity creation is consistently negatively associated with short-term wholesale funding, but not with long-term wholesale funding. Our results suggest that the relationship of short-term wholesale funding with asset-side liquidity creation is significantly driven by a negative relationship with illiquid lending. Moreover, our results show that the negative relationship between wholesale funding and liquidity creation is positively moderated by asset risk, suggesting the presence of moral hazard incentives. Our results are robust to a series of tests and have important implications for bank liquidity regulation.

## Introduction

This paper aims to investigate whether limiting banks’ reliance on wholesale funding affects their main function in the economy, which is creating liquidity. Wholesale funding includes several financing resorts, such as interbank lending, repurchase agreements (repo) or debt securities issued for money market mutual funds, which are used by banks as a supplement to deposits or to expand their balance sheet. The literature has attempted to understand the consequences of reliance on wholesale funding but it remains unclear whether relying on wholesale funding is beneficial. There are many questions remaining unanswered: What is the relationship between wholesale funding and lending after the crisis? What is the relationship between wholesale funding and more comprehensive measures of bank output such as liquidity creation? Our study aims to answer these questions.

Focusing on the post-2009 period, our study provides three main findings. First, we document a consistently negative association of asset-side liquidity creation with short-term wholesale funding, but not with long-term wholesale funding. Second, we find a negative relationship between short-term wholesale funding and illiquid lending. Third, we show that the relationship between wholesale funding and asset-side liquidity creation is moderated by asset risk. Consistent with moral hazard theory, our findings suggest that banks are less likely to adjust their liquidity creation levels as a response to increased funding risk, thus engaging in suboptimal risk-taking. These results are robust to a battery of tests.

Our study makes a novel contribution to the literature on bank liquidity creation. Banks’ ability to fund illiquid loans with liquid deposits is one of their main functions (Bryant 1980; Diamond and Dybvig 1983). Through their unique intermediation skills, banks create liquidity for depositors, who can withdraw their money when they wish at par value, and for borrowers that receive loans to fund their investment projects. Although banks’ liquidity creation function seems to work well during normal times (Acharya and Mora 2015) and contributes positively to real economic output (Berger and Sedunov 2017), using liquid deposits to create illiquid loans leaves banks vulnerable to bank runs (Diamond and Dybvig 1983; Leiva and Mendizábal 2019).

The determinants of bank liquidity creation, however, have not been well explored due to difficulties in measurement. Since Berger and Bouwman (2009) introduced a quantitative method to measure liquidity creation,^1^ attempts have been made to investigate the influential factors of bank liquidity creation, including capital (Distinguin et al. 2013; Horváth et al. 2014; Fungáčová et al. 2017; Casu et al. 2019), market liquidity (Chatterjee 2015), CEO optimism (Huang et al. 2018) and governance (Díaz and Huang 2017). However, so far, the literature has neglected how alternative types of bank funding, such as wholesale funding, are related to liquidity creation, except for a few studies examining how wholesale funding affects lending (e.g. Cornett et al. 2011; Dewally and Shao 2014; Dagher and Kazimov 2015), which is only one element of banks’ liquidity creation function.

Wholesale funding is often raised on a short-term rollover basis, and this leaves banks vulnerable as wholesale financiers may refuse to rollover debt based on superior information on the bank (Calomiris and Kahn 1991) or they may even have low incentives to implement costly monitoring during noisy public signals (Huang and Ratnovski 2011). In times of stress, raising capital appears to be more expensive than it is in ordinary times (Freixas and Rochet 2008), and this leads to the failure of individual banks and even to market failures (Imbierowicz and Rauch 2014; Berger and Bouwman 2017). The 2007–2009 financial crisis highlighted the risk that is inherently associated with banks as liquidity providers. Empirical evidence documents that during the crisis, banks that relied heavily on wholesale funding contracted their lending to mitigate liquidity risk and prevent inefficient liquidations of their assets (e.g. Cornett et al. 2011; Dagher and Kazimov 2015).

Our study also contributes to the understanding of moral hazard in the banking industry. According to moral hazard theory, bank managers may have incentives to increase their risk-taking to suboptimal levels, to enhance their private benefits or to deal with the conflict of interest between shareholders and creditors (Jensen and Meckling 1976). We examine whether the presence of moral hazard through asset risk (i.e. poor asset quality) can affect the relationship between wholesale funding and liquidity creation, leading to suboptimal risk-taking by banks.

Our findings have important policy implications for regulations targeted at increasing bank liquidity buffers. Regulators have been particularly concerned with the adverse effects that wholesale funding may have on bank liquidity after the financial crisis, especially in a highly interconnected global banking system where contagion of risk can be critical for financial stability (Ballester et al. 2016). To protect banks from funding risk, regulators introduced new liquidity requirements under Basel III (i.e. the Liquidity Coverage Ratio (henceforth LCR) and the Net Stable Funding Ratio (henceforth NSFR)), through which banks that rely more on wholesale funding are punished (Banerjee and Mio 2018; Tarullo 2019). It is, however, unclear from the extant literature whether the post-2009 liquidity buffers imposed on banks address effectively the vulnerabilities of the banking system. It is argued that wholesale funding is closely related to banks’ insolvency risk, but views of academics on liquidity regulation are inconsistent. Some researchers consider it an appropriate intervention (e.g. Acharya and Mora 2015), whereas others argue that the 2007–2009 financial crisis was an insolvency risk crisis rather than a liquidity crisis and the current emphasis on liquidity requirements is misplaced (e.g. Thakor 2018). Therefore, by examining the relationship of wholesale funding with banks’ main function in the economy, we add to the discussion on one of the key consequences of liquidity regulation.

The remainder of the paper is structured as follows: Sect. 2 discusses the theoretical framework and hypotheses development; Sect. 3 describes the empirical methodology; Sect. 4 discusses the data, sample, and descriptive statistics; Sect. 5 outlines and discusses the empirical results; Sect. 6 presents the robustness tests; and Sect. 7 concludes and discusses the policy implications.

## Theoretical Framework and Hypotheses Development

In this section, we review the theoretical and empirical literature that helps us formulate the theoretical framework and our expectations for the relationship between wholesale funding and liquidity creation, as well as the moderating role of poor asset quality.

### The positive effect of Wholesale funding on liquidity creation

Under a fragile deposit structure,^2^ banks develop specific collection skills that allow the generation of illiquid loans to honour the commitment of paying back depositors (Diamond 1984; Diamond and Rajan 2001). While banks have traditionally focused on building relationships with borrowers to enhance monitoring and ensure full payment, nowadays, they also evaluate the creditworthiness of borrowers based on quantitative information with the use of internal credit scoring models (Mester 1997; Akhavein et al. 2005). Even though banks have developed various tools to increase monitoring when needed, this seems unable to reduce the default risk associated with the deposit structure.^3^

A fragile deposit structure becomes more vulnerable when deposits are substituted by wholesale funding (Pérignon et al. 2018). Compared with retail depositors, wholesale financiers are better informed about the quality of the bank and are more likely to withdraw their funding in times of adverse signals. These sophisticated investors who have access to superior fundamental information about the condition of the bank are the first to cut financing to obtain greater recovery value when the quality of the bank drops (Calomiris and Kahn 1991).^4^ Also, as Huang and Ratnovski (2011) suggest, when wholesale financiers expect an early liquidation without significant losses based on negative public signals, they are likely to avoid conducting costly private monitoring and may opt to withdraw.^5^ Dewally and Shao (2014) find that in normal periods, wholesale funding has a positive effect on bank lending. Banks with greater reliance on wholesale funding are more likely to operate under the threat of liquidation and they are forced to increase borrower monitoring to avoid any liquidation of their assets. Improved borrower monitoring would then allow banks to extend their lending. At the same time, wholesale financiers may encourage banks to focus their activities on the traditional bank business of creating loans, preventing investments in nonbank activities such as securities underwriting or trading that reduce liquidity creation. Therefore, we formulate Hypothesis 1 based on the expectation of a positive effect on illiquid assets.

**H1.** Wholesale funding helps banks create more liquidity.

### The negative effect of Wholesale Funding on Liquid Creation

Wholesale funding may have a negative effect on illiquid loans and asset-side liquidity creation as well. While wholesale funding offers flexibility to banks’ liability side, it exposes them to market-wide liquidity shocks. The literature documents that during the financial crisis of 2007–2009 banks that relied more on wholesale funding contracted their lending after experiencing liquidity shocks to preserve liquidity (Cornett et al. 2011; Dagher and Kazimov 2015). This lending reduction contributed disproportionally to the contraction in the supply of credit during the crisis. Yet, banks that relied more on deposits and equity capital were able to continue creating loans relative to other banks.

Banks funded largely with insured deposits generally have a greater capacity of creating illiquid loans without exposing themselves to the risk of maturity transformation. On the other hand, banks that rely a greater share of their funding structure on non-deposit funding such as short-term wholesale funding are more exposed to rollover risk which may prevent them from creating illiquid loans. Considering this association of wholesale funding with greater liquidity risk, banks may respond to the increased exposures by hoarding liquid assets such as cash and reserves and by reducing illiquid lending, hence creating less liquidity. Paligorova and Santos (2017) find that banks that rely more on short-term wholesale funding are likely to shorten the maturity of their loans. We therefore formulate Hypothesis 2 as:

**H2.** Wholesale funding prevents banks from creating more liquidity.

### The moderating role of Asset Quality and Moral Hazard

While in H2 we argue that banks may respond to funding risk by contracting their liquidity creation levels, would this be in the interest of both principals and agents who are under different incentives? We posit that the relationship between wholesale funding and liquidity creation can be moderated by the presence of moral hazard.

In a general sense, both investors and regulators are likely to be risk averse when banks rely more on unstable funding. Wholesale financiers want the bank to deliver the project that they have invested in without taking on more risk that jeopardizes their expected returns, while regulators are concerned about the liquidity risk associated with reliance on wholesale funding and want the bank to increase the share of liquid assets to better anticipate liquidity shocks.

However, theory and empirical evidence on moral hazard suggest that bank managers may have incentives to take more risk than the optimal level (Guo et al. 2015) Jensen and Meckling (1976) argue that moral hazard problems can take two forms. First, bank managers may seek private benefits by investing in new pet projects that may be value-reducing for the bank. Second, moral hazard problems may arise because of conflicts of interest between shareholders and creditors such as depositors or wholesale financiers. In this case, the bank may choose to shift the additional suboptimal risk to its creditors. Therefore, in the presence of moral hazard, banks may not adjust their liquidity creation levels due to increased funding risk.

Although moral hazard is not easily and directly observable, poor asset quality is well-documented by earlier research to be strongly associated with moral hazard. One strand of the literature supports the moral hazard hypothesis by showing that poor asset quality increases the risk-taking behaviour of banks (e.g. Nier and Baumann 2006; Zhang et al. 2016). Another strand of the literature suggests that poor asset quality may motivate banks to hide problem loans (e.g. Niinimaki 2012) and misreport the truth (e.g. Flanagan and Purnanandam 2019; Kladakis et al. 2020). In such ways, poor asset quality incentivizes managers to shift the suboptimal risk to the bank’s creditors. We therefore formulate Hypothesis 3 as:

**H3.** Poor asset quality mitigates the negative relationship between wholesale funding and liquidity creation.

## Empirical methodology

### Liquidity creation measures

We follow Berger and Bouwman’s (2009) three-step methodology to construct two asset-side liquidity creation measures.^6^ First, we classify all balance sheet items into three groups according to their level of liquidity, namely liquid, semi-liquid and illiquid. Second, we assign a positive weight (½) to illiquid assets, while we assign a negative weight (-½) to liquid assets. These weights are used consistently with the theory arguing that maximum liquidity is created when illiquid assets are converted into liquid liabilities, while maximum liquidity is destroyed when liquid assets are converted into illiquid liabilities or equity. More specifically, we assign a positive weight to illiquid loans, fixed, intangible and other assets, and a negative weight to cash and cash balances, securities and trading assets. These classifications are based on the ease, cost, and time for banks to liquidate their obligations and meet liquidity demand on the liability side. For instance, it is more difficult for banks to liquidate their fixed than their trading assets to satisfy the liquidity demand by depositors. Assets classified as semi-liquid are given a zero weight and are not included in the calculation of the liquidity creation measures. Third, the liquidity measures are constructed by putting together the weighted balance sheet items. Two asset-side liquidity creation measures are constructed (ASLC1 and ASLC2), as we use two measures for illiquid loans (corporate loans and long-term loans). The final measures are normalised by total assets.

### Baseline regression Framework

Our baseline analysis of the relationship between wholesale funding and liquidity creation is conducted using fixed-effects regressions in the following form:

1 $${Liquidity Creation}_{i,t}= {\alpha }_{i}+{{\beta }_{1}Wholesale Funding}_{i,t}+ \sum _{j=1}^{7}{\beta }_{j} {Bank Control}_{i,t}+{\lambda }_{t}+{\epsilon }_{i,t}$$

where i = 1, …, N indexes the bank, t indexes the year of the observation, ${\alpha }_{i}$ is the bank-level fixed-effect, ${\lambda }_{t}$ is the time effect for year t, while ${\epsilon }_{i,t}$ is the error term, assumed to be normally distributed with mean 0 and variance ${\sigma }^{2}$. We use bank fixed-effects to control for differences among the banks that cannot be captured by the control variables and to alleviate correlations across error terms. The inclusion of bank fixed-effects is supported by the Hausman test. We also use year fixed effects to account for serial correlation and to eliminate bias from unobservables that change over time but are constant over banks. Finally, following the literature we run the regressions using robust standard errors clustered at the bank level to control for heteroskedasticity.

${Liquidity Creation}_{i,t}$ is one of the two asset-side liquidity measures (ASLC1 or ASLC2) as constructed in Sect. 3.1. ${Wholesale Funding}_{i,t}$ is either short- or long-term wholesale funding (WFST or WFLT). We include seven bank-level control variables often used in the literature that examines the determinants of liquidity creation (e.g. Berger and Bouwman 2009; Berger et al. 2016; Zheng et al. 2019). First, PL stands for problem loans normalized by net total loans. The deterioration of asset quality and increase in credit risk is likely to impede liquidity creation. Second, EQRAT stands for the equity to total assets ratio and we could expect either a negative or a positive relationship consistent with the financial fragility and risk absorption hypotheses, respectively. Third, ROAA stands for the return on average assets and we could expect that greater profitability would enable liquidity creation. Fourth, LNTA stands for the natural logarithm of total assets and we could expect either a positive or a negative relationship with liquidity creation. While larger banks may exploit greater economies of scale to increase their liquidity creation, they are also subject to more regulatory scrutiny that may prevent them from taking more risk. Fifth, LNZSCORE stands for the natural logarithm of the Z-SCORE and higher overall bank stability should enable liquidity creation. Sixth, MQ stands for the ratio of operating expenses to operating income and it is a measure of operating inefficiency that could harm liquidity creation. Finally, NIM stands for the net interest income, a measure of overall bank efficiency that is likely to increase the capacity of banks to create liquidity.

## Data, sample and descriptive analysis

We use bank-level data based on consolidated financial statements to construct all variables used in our baseline regressions. Data is obtained from the S&P Global Market Intelligence database. Some macroeconomic data used in our robustness tests is obtained from the International Monetary Fund. The data has an annual frequency, and our analysis focuses on the period of 2010–2020.^7^ Similar to Demirgüç-Kunt and Huizinga (2010), Dewally and Shao (2014) and Acharya and Mora (2015), we use total wholesale funding measures which include all financial liabilities and repurchase agreements except for derivatives and customer deposits. In contrast to related studies that use individual components of wholesale funding such as certificates of deposits (CDs) (Pérignon et al. 2018) and repurchase agreements (repo) (De Haan and Van Den End 2013), this allows us to examine the total effects of reliance on wholesale funding. The calculation of short- and long-term wholesale funding is provided by the S&P Global Market Intelligence database, and we normalize each one by total assets. WFST contains the liabilities with maturity less than one year and WFLT the liabilities with maturity greater than or equal to one year.

The sample contains a diverse group of 825 banks from 84 countries. Panel A in Table 1 presents the distribution of asset-side liquidity creation observations across regions. About half of the observations are from European banks, and about a third of the observations are from banks in the Asia-Pacific region. Indicatively, the biggest contributors to asset-side liquidity creation observations are China and France, followed by the United Kingdom and Japan. Our sample of liquidity creation observations is similar to what other studies have used in the literature. For instance, Kladakis et al. (2021) use the same database to construct liquidity creation measures mainly for banks from Europe and Asia-Pacific, while Fu et al. (2016) focus on Asia-Pacific and Casu et al. (2019) on Eurozone countries.

**Table 1:** Panel A: Distribution of Asset-Side Liquidity Creation Observations Across Regions

Region	ALC1 Obs.		Perc.		ALC2 Obs.		Perc.		Common Obs.			
Europe	1605		49%		1931		50%		1293			
Asia-Pacific	1058		33%		1508		39%		915			
Latin America and Caribbean	349		11%		180		5%		179			
Middle East	178		5%		168		4%		136			
Africa	27		1%		35		1%		17			
North America (Canada)	35		1%		25		1%		22			
Panel B: Summary Descriptive Statistics.												
		Obs.		Median		Mean		Std. Dev.		5th Perc.		95th Perc.
ALC1		3252		0.004		0.016		0.129		-0.216		0.199
ALC2		3847		0.062		0.076		0.176		-0.245		0.301
ALC1-B		3252		-0.011		0.001		0.129		-0.229		0.180
ALC2-B		3847		0.047		0.057		0.177		-0.262		0.290
ALC1-OBS		2277		0.088		0.081		0.152		-0.141		0.332
ALC2-OBS		2409		0.138		0.155		0.189		-0.171		0.389
CLOANS		3252		0.303		0.289		0.154		0.058		0.580
LTLOANS		3847		0.408		0.411		0.212		0.077		0.719
WFST		4537		0.127		0.095		0.112		0.007		0.331
WFLT		4537		0.112		0.066		0.125		0.000		0.360
PL		4537		0.054		0.025		0.092		0.003		0.231
LLR		4537		0.033		0.022		0.039		0.002		0.109
EQRAT		4537		0.090		0.082		0.043		0.039		0.163
ROAA		4537		0.008		0.007		0.010		-0.003		0.023
LNTA		4537		16.829		16.711		1.730		13.941		20.078
LNZSCORE		4537		3.130		3.192		0.931		1.478		4.468
NIM		4537		0.024		0.020		0.018		0.006		0.058
MQ		4537		0.571		0.553		0.221		0.316		0.852
GDP		4537		0.031		0.024		0.031		-0.009		0.079
UNEMP		4537		0.064		0.050		0.047		0.000		0.137
INF		4537		0.024		0.019		0.031		-0.004		0.075

The descriptive statistics refer to the variables in the form used in the regressions. The number of observations refers to the independent variables’ common sample when at least one of the dependent variables is available

**Fig. 1:**
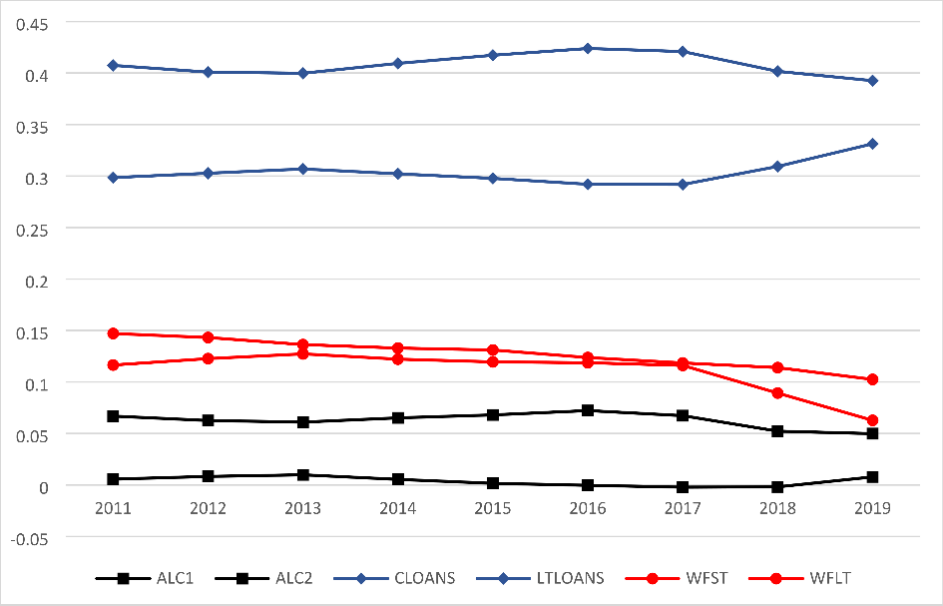
The evolution of wholesale funding, asset-side liquidity creation and illiquid lending. The figure presents the two-year moving average of all variables

Figure 1 presents the two-year moving average of our wholesale funding, asset-side liquidity creation and illiquid lending. We observe that banks continuously rely more on WFST than on WFLT. Also, the graph shows that at the time of the proposal of the regulatory liquidity ratios in 2010, banks obtained more than 30% of their funding structure from wholesale financiers. This heavy reliance on wholesale funding gradually decreased in the subsequent years as banks were slowly adjusting their liquidity exposures to the new regulatory requirements. Moreover, we do not observe such significant changes in liquidity creation levels over time. Figure 1 shows that ALC1 slightly reduced over time but increased in the last years, which is mainly driven by its key component CLOANS. However, ALC2 marginally decreased and LTLOANS remained relatively stable over the sample period.

## Empirical results and discussion

### Baseline regressions

Our baseline results are presented in Tables 2 and 3 which report the regression estimates on the relationship between asset-side liquidity creation and short- and long-term wholesale funding (WFST and WFLT, respectively). In both tables, we first present the regressions without using the control variables that we introduce later. We observe that the coefficients of WFST are negative and significant at the 1% level throughout all regressions in Table 2. These relationships are also economically significant, as a one standard deviation increase in WFST is associated with a decrease in asset-side liquidity creation by approximately 2-2.6% points and a decrease in illiquid lending by approximately 1.7–2.1% points. On the contrary and consistent with our expectations, we observe that the relationship between asset-side liquidity creation and WFLT is not statistically significant except for the regression in column (5) of Table 3 where the coefficient of WFLT is positive and significant at the 5% level.

**Table 2:** The relationship between short-term wholesale funding and asset-side liquidity creation

	(1)	(2)	(3)	(4)	(5)	(6)	(7)	(8)
	ALC1	CLOANS	ALC2	LTLOANS	ALC1	CLOANS	ALC2	LTLOANS
WFST	-0.255***	-0.187***	-0.199***	-0.137***	-0.233***	-0.192***	-0.177***	-0.156***
	(0.059)	(0.047)	(0.028)	(0.038)	(0.060)	(0.050)	(0.031)	(0.040)
PL					-0.058	-0.080	-0.042	-0.009
					(0.045)	(0.054)	(0.046)	(0.044)
EQRAT					0.268	0.394*	-0.246	-0.724***
					(0.195)	(0.219)	(0.160)	(0.184)
ROAA					-0.176	-0.360	-0.194	-0.383
					(0.257)	(0.293)	(0.228)	(0.274)
LNTA					-0.003	0.009	-0.006	0.020**
					(0.010)	(0.012)	(0.008)	(0.010)
LNZSCORE					-0.007	-0.026	0.022	0.051***
					(0.020)	(0.023)	(0.014)	(0.016)
NIM					1.014***	0.602*	1.664***	1.506***
					(0.336)	(0.307)	(0.273)	(0.466)
MQ					-0.013	-0.031**	0.007	-0.006
					(0.012)	(0.013)	(0.013)	(0.012)
CONSTANT	0.053***	0.346***	0.089***	0.418***	0.071	0.253	0.088	-0.037
	(0.008)	(0.007)	(0.005)	(0.007)	(0.166)	(0.195)	(0.146)	(0.182)
Bank FE	YES	YES	YES	YES	YES	YES	YES	YES
Time FE	YES	YES	YES	YES	YES	YES	YES	YES
Obs.	3,122	3,122	3,707	3,707	3,122	3,122	3,707	3,707
N. of Banks	592	592	712	712	592	592	712	712
R2 - Within	0.106	0.074	0.074	0.080	0.133	0.094	0.111	0.122

The dependent variables are ALC1, ALC2, CLOANS and LTLOANS. ALC1 and ALC2 refer to asset-side liquidity creation containing corporate and long-term loans, respectively. CLOANS and LTLOANS are the corporate and long-term loans ratios, respectively. WFST stands for short-term wholesale funding. PL is problem loans. EQRAT is the equity ratio. ROAA is the return on average assets. LNTA is the natural logarithm of total assets in Euros. LNZSCORE is the natural logarithm of the ZSCORE. NIM is the net interest margin. MQ is the cost-to-income ratio. The regressions include bank and time fixed-effects. Robust standard errors clustered at the bank level are reported in parentheses. *, ** and *** denote significance at the 10%, 5%, and 1% level, respectively

**Table 3:** The relationship between long-term wholesale funding and asset-side liquidity creation

	(1)	(2)	(3)	(4)	(5)	(6)	(7)	(8)
	ALC1	CLOANS	ALC2	LTLOANS	ALC1	CLOANS	ALC2	LTLOANS
WFLT	0.054	0.009	-0.009	0.033	0.100**	0.021	0.007	0.012
	(0.038)	(0.052)	(0.043)	(0.044)	(0.039)	(0.053)	(0.045)	(0.043)
PL					-0.085**	-0.098*	-0.048	-0.013
					(0.043)	(0.055)	(0.042)	(0.042)
EQRAT					0.269	0.399*	-0.216	-0.696***
					(0.203)	(0.216)	(0.170)	(0.185)
ROAA					-0.200	-0.368	-0.189	-0.377
					(0.266)	(0.295)	(0.244)	(0.288)
LNTA					-0.021**	-0.005	-0.015*	0.012
					(0.009)	(0.011)	(0.008)	(0.010)
LNZSCORE					0.001	-0.023	0.023	0.052***
					(0.019)	(0.022)	(0.015)	(0.016)
NIM					1.100***	0.679**	1.781***	1.608***
					(0.370)	(0.326)	(0.283)	(0.475)
MQ					-0.007	-0.027**	0.010	-0.003
					(0.011)	(0.013)	(0.013)	(0.013)
CONSTANT	0.010	0.317***	0.063***	0.395***	0.302*	0.440**	0.201	0.063
	(0.007)	(0.008)	(0.007)	(0.007)	(0.162)	(0.192)	(0.150)	(0.182)
Bank FE	YES	YES	YES	YES	YES	YES	YES	YES
Time FE	YES	YES	YES	YES	YES	YES	YES	YES
Obs.	3,122	3,122	3,707	3,707	3,122	3,122	3,707	3,707
N. of Banks	592	592	712	712	592	592	712	712
R2 - Within	0.024	0.032	0.023	0.065	0.077	0.055	0.074	0.104

The dependent variables are ALC1, ALC2, CLOANS and LTLOANS. ALC1 and ALC2 refer to asset-side liquidity creation containing corporate and long-term loans, respectively. CLOANS and LTLOANS are the corporate and long-term loans ratios, respectively. WFLT stands for long-term wholesale funding. PL is problem loans. EQRAT is the equity ratio. ROAA is the return on average assets. LNTA is the natural logarithm of total assets in Euros. LNZSCORE is the natural logarithm of the ZSCORE. NIM is the net interest margin. MQ is the cost-to-income ratio. The regressions include bank and time fixed-effects. Robust standard errors clustered at the bank level are reported in parentheses. *, ** and *** denote significance at the 10%, 5%, and 1% level, respectively

Two main findings emerge from our baseline regressions. First, in line with hypothesis 2, we find a negative relationship between wholesale funding and liquidity creation, and such a negative relationship is significantly driven by the short-term component of wholesale funding rather than the long-term component of it. This is consistent with recent literature that reveals the risks associated with unstable market-based funding (e.g. Dermirguc-Kunt and Huizinga 2010; Huang and Ratnovski 2011; López-Espinosa et al. 2012; Ahnert et al. 2019).^8^ It appears that although reliance on wholesale funding may force banks to monitor their borrowers more heavily, banks are more concerned about their exposure to liquidity shocks in the short-run. This is a novel finding that encourages regulators to place greater emphasis on the effects of short-term wholesale funding on more comprehensive measures of bank output such as liquidity creation. For instance, regardless of the effect of wholesale funding on illiquid lending, an increase in cash, reserves and other liquid assets also reduces liquidity creation. In this case, short-term wholesale funding can adversely and significantly affect the level of liquidity created in the economy.

Second, the results suggest that the negative effect of wholesale funding on the supply of credit is not unique to the crisis (Cornett et al. 2011; Dagher and Kazimov 2015) and that it holds in the more normal post-2009 period examined using this sample. It is neither consistent with the finding by Dewally and Shao (2014) who document a positive effect of wholesale funding on lending in the pre-crisis period. This documented change in bank behaviour could be attributed to the introduction of greater liquidity requirements that possibly make banks more cautious when considering their liquidity exposures.

### The moderating role of Asset Quality and Moral Hazard

We extend our analysis to test whether poor asset quality can mitigate the negative relationship between wholesale funding and liquidity creation (H3). We introduce an interaction term between poor asset quality, measured by problem loans (PL) and WFST to our baseline regression models.^9^ The results of these regressions are presented in Table 4 and are consistent with our expectations of H3. First, the interaction term between WFST and PL is positive and highly significant across all regressions. This suggests that under greater asset risk the relationship between WFST and liquidity creation is positively moderated. Second, we observe that the coefficient of WFST is greater in columns (5) to (8) compared to the respective coefficients in Table 3. This suggests that when PL is zero banks make greater adjustments to their liquidity creation levels as a response to higher funding risk.

**Table 4:** The moderating role of asset quality on the relationship between wholesale funding and asset-side liquidity creation

	(1)	(2)	(3)	(4)	(5)	(6)	(7)	(8)
	ALC1	CLOANS	ALC2	LTLOANS	ALC1	CLOANS	ALC2	LTLOANS
WFST	-0.307***	-0.230***	-0.245***	-0.177***	-0.281***	-0.234***	-0.217***	-0.195***
	(0.066)	(0.050)	(0.031)	(0.042)	(0.067)	(0.054)	(0.034)	(0.044)
PL	-0.153***	-0.132**	-0.191***	-0.193***	-0.155***	-0.165***	-0.152***	-0.113**
	(0.054)	(0.060)	(0.051)	(0.054)	(0.057)	(0.064)	(0.058)	(0.058)
WFST*PL	0.912***	0.751**	0.877***	0.755***	0.794***	0.695**	0.747***	0.714***
	(0.284)	(0.339)	(0.164)	(0.188)	(0.263)	(0.296)	(0.193)	(0.219)
Control variables	NO	NO	NO	NO	YES	YES	YES	YES
Bank FE	YES	YES	YES	YES	YES	YES	YES	YES
Time FE	YES	YES	YES	YES	YES	YES	YES	YES
Obs.	3,122	3,122	3,707	3,707	3,122	3,122	3,707	3,707
N. of Banks	592	592	712	712	592	592	712	712
R2 - Within	0.121	0.084	0.088	0.088	0.144	0.102	0.119	0.127

The dependent variables are ALC1, ALC2, CLOANS and LTLOANS. ALC1 and ALC2 refer to asset-side liquidity creation containing corporate and long-term loans, respectively. CLOANS and LTLOANS are the corporate and long-term loans ratios, respectively. WFST stands for short-term wholesale funding. PL is problem loans. The same control variables as in Table 2 are used. The regressions include bank and time fixed-effects. Robust standard errors clustered at the bank level are reported in parentheses. *, ** and *** denote significance at the 10%, 5%, and 1% level, respectively

The positive moderation of asset risk on the negative relationship between WFST and asset-side liquidity creation can be attributed to the presence of moral hazard incentives. The literature argues that asset risk increases the risk-taking behaviour of banks due to conflicts of interest between shareholders and creditors (e.g. Zhang et al. 2016). Our results indicate that this is the case as banks do not significantly adjust their liquidity creation levels to protect themselves from funding risk. Instead, with greater reliance on wholesale funding and higher exposure to asset risk, they shift the suboptimal risk to their creditors such as depositors and wholesale financiers.

## Robustness and endogeneity tests

We conduct a series of robustness tests to ensure the soundness of our findings presented in the previous section. For brevity, we focus on our key findings of the direct negative relationship between short-term wholesale funding and liquidity creation and the moderating role of asset quality.

### Alternative measures of asset-side liquidity creation and Asset Quality

We initially test whether our results hold when using alternative measures of asset-side liquidity creation and asset quality. First, one might have concerns about the inclusion of fixed, intangible and other assets in our main asset-side liquidity creation variables since these balance sheet items do not create liquidity in the market on their own. Therefore, we remove these items from our calculation and create two new variables ASLC1-B and ASLC2-B. Second, due to limited data availability, our baseline asset-side liquidity creation measures do not include any off-balance sheet items which can at times largely exceed in size the on-balance sheet items. To partially address this issue, we include credit commitments after assigning a positive weight of 0.5 according to Berger and Bouwman (2009). Our variables that include credit commitments are denoted as ASLC1-OBS and ASLC2-OBS. The results using the alternative asset-side liquidity creation variables are presented in Table 5 and the coefficients of WFST and of the interaction term with PL maintain their signs and are highly significant.

**Table 5:** The relationship between short-term wholesale funding and asset-side liquidity creation using alternative measures of liquidity creation

	(1)	(2)	(3)	(4)	(5)	(6)	(7)	(8)
	ALC1-B	ALC2-B	ALC1-B	ALC2-B	ALC1-OBS	ALC2-OBS	ALC1-OBS	ALC2-OBS
WFST	-0.230***	-0.178***	-0.279***	-0.219***	-0.398***	-0.325***	-0.431***	-0.368***
	(0.059)	(0.031)	(0.066)	(0.035)	(0.079)	(0.044)	(0.084)	(0.048)
PL	-0.055	-0.033	-0.153***	-0.142***	-0.150	0.037	-0.311***	-0.183*
	(0.044)	(0.045)	(0.056)	(0.058)	(0.096)	(0.095)	(0.114)	(0.109)
WFST*PL			0.804***	0.745***			0.925***	1.572***
			(0.260)	(0.198)			(0.343)	(0.481)
Control variables	YES	YES	YES	YES	YES	YES	YES	YES
Bank FE	YES	YES	YES	YES	YES	YES	YES	YES
Time FE	YES	YES	YES	YES	YES	YES	YES	YES
Obs.	3,122	3,707	3,122	3,707	2,176	2,291	2,176	2,291
N. of Banks	592	712	592	712	557	611	557	611
R2 - Within	0.129	0.113	0.141	0.121	0.253	0.206	0.259	0.214

The dependent variables are ALC1-B, ALC2-B, ALC1-OBS and ALC2-OBS. ALC1-B and ALC2-B are the same variables as ALC1 and ALC2 but excluding fixed, intangible and other assets. ALC1-OBS and ALC2-OBS are the same variables as ALC1 and ALC2 but including credit commitments weighted by 0.5. WFST stands for short-term wholesale funding. PL is problem loans. The same control variables as in Table 2 are used. The regressions include bank and time fixed-effects. Robust standard errors clustered at the bank level are reported in parentheses. *, ** and *** denote significance at the 10%, 5%, and 1% level, respectively

Since our analysis emphasizes the moderating role of bank asset quality, we also test our results using an alternative measure of bank asset quality. More specifically, in this test, we use loan loss reserves (LLR) instead of PL. LLR stands for the ratio of loan loss reserves to total loans and leases. The results are reported in Table 6 and largely confirm our findings.

**Table 6:** The moderating role of asset quality on the relationship between wholesale funding and asset-side liquidity creation using an alternative measure of asset quality

	(1)	(2)	(3)	(4)	(5)	(6)	(7)	(8)
	ALC1	CLOANS	ALC2	LTLOANS	ALC1	CLOANS	ALC2	LTLOANS
WFST	-0.230***	-0.188***	-0.178***	-0.156***	-0.285***	-0.235***	-0.247***	-0.232***
	(0.059)	(0.049)	(0.031)	(0.040)	(0.067)	(0.059)	(0.043)	(0.051)
LLR	-0.353**	-0.415**	-0.335**	-0.107	-0.498***	-0.537***	-0.539***	-0.331**
	(0.136)	(0.165)	(0.136)	(0.139)	(0.170)	(0.206)	(0.148)	(0.159)
WFST*LLR					1.492**	1.258*	2.218***	2.430***
					(0.656)	(0.755)	(0.772)	(0.808)
Control variables	YES	YES	YES	YES	YES	YES	YES	YES
Bank FE	YES	YES	YES	YES	YES	YES	YES	YES
Time FE	YES	YES	YES	YES	YES	YES	YES	YES
Obs.	3,122	3,122	3,707	3,707	3,122	3,122	3,707	3,707
N. of Banks	592	592	712	712	592	592	712	712
R2 - Within	0.143	0.106	0.118	0.123	0.150	0.110	0.126	0.129

The dependent variables are ALC1, ALC2, CLOANS and LTLOANS. ALC1 and ALC2 refer to asset-side liquidity creation containing corporate and long-term loans, respectively. CLOANS and LTLOANS are the corporate and long-term loans ratios, respectively. WFST stands for short-term wholesale funding. LLR is loan loss reserves. The same control variables as in Table 2 are used. The regressions include bank and time fixed-effects. Robust standard errors clustered at the bank level are reported in parentheses. *, ** and *** denote significance at the 10%, 5%, and 1% level, respectively

### Endogeneity Issues

An important issue is that changes in bank liquidity creation could make banks adjust their wholesale funding structure, raising endogeneity concerns for our regressions. We employ two tests to address this. First, we run the regressions using the independent variables in a lagged form.^10^ More specifically, we use the average of the first two lags of our independent variables. The results of this test are presented in Table 7. We observe that the coefficients maintain their signs and significance, confirming our initial results.^11^

**Table 7:** The relationship between short-term wholesale funding and asset-side liquidity creation using the average of the first two lags of the independent variables

	(1)	(2)	(3)	(4)	(5)	(6)	(7)	(8)
	ALC1	CLOANS	ALC2	LTLOANS	ALC1	CLOANS	ALC2	LTLOANS
WFST	-0.251***	-0.201***	-0.131***	-0.097*	-0.301***	-0.250***	-0.168***	-0.145***
	(0.077)	(0.069)	(0.034)	(0.050)	(0.087)	(0.078)	(0.038)	(0.056)
PL	-0.171**	-0.250***	-0.040	-0.020	-0.286***	-0.362***	-0.149	-0.160*
	(0.078)	(0.071)	(0.065)	(0.060)	(0.106)	(0.091)	(0.098)	(0.087)
WFST*PL					1.189**	1.153**	0.949**	1.222***
					(0.531)	(0.519)	(0.379)	(0.433)
Control variables	YES	YES	YES	YES	YES	YES	YES	YES
Bank FE	YES	YES	YES	YES	YES	YES	YES	YES
Time FE	YES	YES	YES	YES	YES	YES	YES	YES
Obs.	2,141	2,141	2,526	2,526	2,141	2,141	2,526	2,526
N. of Banks	474	474	560	560	474	474	560	560
R2 - Within	0.120	0.110	0.058	0.071	0.130	0.119	0.063	0.076

The dependent variables are ALC1, ALC2, CLOANS and LTLOANS. ALC1 and ALC2 refer to asset-side liquidity creation containing corporate and long-term loans, respectively. CLOANS and LTLOANS are the corporate and long-term loans ratios, respectively. WFST stands for short-term wholesale funding. PL is problem loans. The same control variables as in Table 2 are used. All independent variables are used with the average of their first two lags. The regressions include bank and time fixed-effects. Robust standard errors clustered at the bank level are reported in parentheses. *, ** and *** denote significance at the 10%, 5%, and 1% level, respectively

Second, we employ the two-step System GMM estimator^12^ to further address endogeneity concerns. One limitation of the fixed-effects estimator is that we cannot be confident that the regressors and the error term are not correlated leading to the endogeneity problem. We use the GMM estimator because it controls for fixed effects, while it avoids the tendency of the fixed-effects transformation to make every observation of the dependent variable endogenous to every other for a given group. We avoid using the difference GMM estimator because we use an unbalanced panel, and we would lose more observations. Instead, the system GMM constructs a system of two equations: the original equation and the first-difference transformation equation which maximizes the number of observations. It is designed for panels with small T and large N, which is the case for our sample, and can help us alleviate problems with possible Nickel bias. Standard errors are usually downward biased in the two-step estimation, so we use Windmeijer’s (2005) finite-sample correction.

Since the system GMM is a complicated estimator that can easily generate invalid results, we are careful and follow the guidelines of Roodman (2009) to increase the likelihood of producing sound estimates. First, we include time dummies to alleviate autocorrelation concerns. Second, we use orthogonal deviations to maximize our sample size since our sample has some gaps. Third, we include all regressors in our instrument matrix. More specifically, we treat liquidity creation, wholesale funding and problem loans as endogenous variables, while we treat our control variables and year dummies as exogenous. Treating our key bank-specific variables as endogenous allows us to use their second and longer lags as instruments (Blundell and Bond 1998). Finally, we make sure that our instrument count is not too high. As basic indications, our instrument count is not higher than the number of banks in a regression and we do not report a perfect p-value of the Hansen statistic of 1.000.

To test the reliability of our results estimated with the system GMM we conduct two specification tests. First, we use Arellano and Bond’s (1991) test for second order autocorrelation in the idiosyncratic disturbance term which could indicate that some lags are not valid as instruments. Second, we use the Hansen J test for overidentifying restrictions to further test the validity of our instruments.

The results of the two-step System GMM estimator are presented in Table 8. The selection of the estimator is supported by the statistically significant coefficient of the lag of the dependent variables, indicating their partial adjustment. Additionally, the Arellano and Bond (1991) test for second order autocorrelation and the Hansen J test for overidentifying restrictions are reported, confirming the soundness of the models. The coefficient of WFST remains negative and significant in all regressions except for when LTLOANS is the dependent variable. Moreover, the coefficient of the interaction term is positive and significant in columns (7) and (8) confirming our baseline results.

**Table 8:** The relationship between short-term wholesale funding and asset-side liquidity creation using the System GMM estimator

	(1)	(2)	(3)	(4)	(5)	(6)	(7)	(8)
	ALC1	CLOANS	ALC2	LTLOANS	ALC1	CLOANS	ALC2	LTLOANS
L.Dependent Var	0.801***	0.900***	0.873***	0.887***	0.768***	0.856***	0.869***	0.874***
	(0.037)	(0.032)	(0.034)	(0.027)	(0.036)	(0.034)	(0.030)	(0.024)
WFST	-0.113**	-0.057**	-0.076*	0.030	-0.139***	-0.068**	-0.110***	-0.017
	(0.047)	(0.029)	(0.042)	(0.063)	(0.048)	(0.030)	(0.039)	(0.053)
PL	-0.029	-0.038	0.035	0.052	-0.014	-0.020	-0.070*	-0.038
	(0.049)	(0.047)	(0.049)	(0.043)	(0.051)	(0.044)	(0.036)	(0.037)
WF*PL					0.209	0.104	0.745**	0.698*
					(0.279)	(0.133)	(0.333)	(0.423)
Control Variables	YES	YES	YES	YES	YES	YES	YES	YES
Year Dummies	YES	YES	YES	YES	YES	YES	YES	YES
Obs.	2509	2509	2949	2949	2509	2509	2949	2949
N. of Banks	496	496	598	598	496	496	598	598
AR(2)	0.330	0.500	0.374	0.610	0.336	0.497	0.376	0.613
Hansen J	0.795	0.494	0.813	0.707	0.997	0.862	0.988	0.989
Instruments	271	271	304	304	356	356	400	400

The dependent variables are ALC1, ALC2, CLOANS and LTLOANS. ALC1 and ALC2 refer to asset-side liquidity creation containing corporate and long-term loans, respectively. CLOANS and LTLOANS are the corporate and long-term loans ratios, respectively. WFST stands for short-term wholesale funding. PL is problem loans. The same control variables as in Table 2 are used. The regressions are ran using the twostep System GMM estimator and include time fixed-effects. Robust standard errors clustered at the bank level are reported in parentheses. *, ** and *** denote significance at the 10%, 5%, and 1% level, respectively

### Sub-Sample Analysis and Macroeconomic Environment

The gradual reduction of banks’ reliance on wholesale funding can largely be attributed to the post-2009 liquidity requirements recommended by the Basel Committee. The banks of the member states of BCBS are subject to the mandatory implementation of these liquidity requirements (i.e. the LCR and NSFR ratios) (Bank for International Settlements (BIS) 2013).^13^ These banks follow a more homogeneous adoption of the liquidity ratios and thus it is important to examine whether the relationship between wholesale funding and liquidity creation holds in this sample as well as to account for different regulation approaches at the country level. We therefore exclude banks from countries that are not BCBS members and are at different levels of regulatory oversight which reduces the sample size by about 40%. We report these results in Table 9 and our findings are confirmed.

**Table 9:** The relationship between short-term wholesale funding and asset-side liquidity creation using banks from BCBS member countries

	(1)	(2)	(3)	(4)	(5)	(6)	(7)	(8)
	ALC1	CLOANS	ALC2	LTLOANS	ALC1	CLOANS	ALC2	LTLOANS
WFST	-0.311***	-0.211***	-0.181***	-0.104**	-0.413***	-0.322***	-0.222***	-0.125***
	(0.088)	(0.059)	(0.037)	(0.043)	(0.102)	(0.062)	(0.042)	(0.046)
PL	-0.159	-0.136	-0.023	0.050	-0.600***	-0.616**	-0.214***	-0.047
	(0.131)	(0.202)	(0.062)	(0.056)	(0.184)	(0.252)	(0.080)	(0.108)
WFST*PL					2.988***	3.255***	0.864***	0.438
					(0.978)	(1.008)	(0.243)	(0.297)
Control variables	YES	YES	YES	YES	YES	YES	YES	YES
Bank FE	YES	YES	YES	YES	YES	YES	YES	YES
Time FE	YES	YES	YES	YES	YES	YES	YES	YES
Obs.	1,703	1,703	2,449	2,449	1,703	1,703	2,449	2,449
N. of Banks	301	301	457	457	301	301	457	457
R2 - Within	0.230	0.146	0.144	0.098	0.252	0.175	0.152	0.099

The dependent variables are ALC1, ALC2, CLOANS and LTLOANS. ALC1 and ALC2 refer to asset-side liquidity creation containing corporate and long-term loans, respectively. CLOANS and LTLOANS are the corporate and long-term loans ratios, respectively. WFST stands for short-term wholesale funding. PL is problem loans. The same control variables as in Table 2 are used. The regressions include bank and time fixed-effects. The sample includes only banks from BCBS member countries. Robust standard errors clustered at the bank level are reported in parentheses. *, ** and *** denote significance at the 10%, 5%, and 1% level, respectively

We also use two additional approaches to account for differences of banks and banking systems across the large number of jurisdictions in our sample. First, we run the regressions using robust standard errors clustered at the country level to control for heteroskedasticity as well as for correlation across observations of the same country in different years and include macroeconomic variables. Our country-level control variables are GDP that stands for real GDP growth, UNEMP that stands for the unemployment rate and INF that stands for the inflation of the country in which each bank is based. Second, we include country dummies and their interaction with the year dummies to control for heterogeneity across jurisdictions in the sample. In both tests presented in Table 10, the results are largely confirmed except for in column (6) where the dependent variable is CLOANS.

**Table 10:** The relationship between short-term wholesale funding and asset-side liquidity creation controlling for the macro environment

	(1)	(2)	(3)	(4)	(5)	(6)	(7)	(8)
	ALC1	CLOANS	ALC2	LTLOANS	ALC1	CLOANS	ALC2	LTLOANS
WFST	-0.287***	-0.235***	-0.214***	-0.189***	-0.242***	-0.023	-0.518***	-0.481***
	(0.075)	(0.073)	(0.041)	(0.063)	(0.036)	(0.037)	(0.039)	(0.044)
PL	-0.166**	-0.175**	-0.158**	-0.116**	-0.068	0.041	-0.125	-0.047
	(0.070)	(0.081)	(0.067)	(0.053)	(0.065)	(0.081)	(0.093)	(0.103)
WFST*PL	0.784**	0.646**	0.751***	0.705***	0.887***	0.223	1.822***	1.048**
	(0.330)	(0.324)	(0.238)	(0.268)	(0.333)	(0.372)	(0.443)	(0.475)
GDP	-0.048	-0.062	0.086	0.011	0.566	0.070	-8.665***	-14.412***
	(0.086)	(0.082)	(0.082)	(0.087)	(1.076)	(0.757)	(2.192)	(3.916)
UNEMP	0.155	0.288*	0.087	0.086	8.035	11.651**	13.551***	18.323***
	(0.156)	(0.154)	(0.158)	(0.153)	(4.976)	(3.917)	(2.717)	(3.428)
INF	0.056	-0.032	0.000	-0.079	1.629	0.955	5.146**	2.949
	(0.068)	(0.070)	(0.067)	(0.119)	(2.694)	(2.573)	(1.991)	(2.024)
Control variables	YES	YES	YES	YES	YES	YES	YES	YES
Bank FE	YES	YES	YES	YES	NO	NO	NO	NO
Time FE	YES	YES	YES	YES	YES	YES	YES	YES
Country*Time Dummies	NO	NO	NO	NO	YES	YES	YES	YES
Obs.	3122	3122	3707	3707	3122	3122	3707	3707
N. of Banks	592	592	712	712	592	592	712	712
R2	0.149	0.111	0.121	0.128	0.571	0.535	0.540	0.556

The dependent variables are ALC1, ALC2, CLOANS and LTLOANS. ALC1 and ALC2 refer to asset-side liquidity creation containing corporate and long-term loans, respectively. CLOANS and LTLOANS are the corporate and long-term loans ratios, respectively. WFST stands for short-term wholesale funding. PL is problem loans. GDP is the real GDP growth of the host country. UNEMP is the unemployment rate of the host country. INF is the inflation growth of the host country. The same control variables as in Table 2 are used. In columns (1) to (4), the regressions include bank and time fixed-effects, while standard errors are clustered at the country level. In columns (5) to (8), the regressions include country and time fixed-effects as well as their interaction, while standard errors are clustered at the bank level. *, ** and *** denote significance at the 10%, 5%, and 1% level, respectively

## Conclusions and policy implications

Banks resort to wholesale funding to finance themselves as a supplement to customer deposits. Although the prevailing view on wholesale funding before the financial crisis of 2007–2009 was that it benefits banks by increasing borrower monitoring under the threat of liquidation, banks’ vulnerability during the crisis has raised concerns about the risk associated with wholesale funding. In this paper, we aim to examine the effect of wholesale funding on asset-side liquidity creation, the most valuable function of banks in the economy.

Against the prevailing view on wholesale funding before the crisis but consistent with recent criticism, our results indicate that wholesale funding is negatively associated with asset-side liquidity creation and its main component, illiquid lending. Such a relationship is mainly attributable to short-term wholesale funding. These results have important implications for regulatory policies aimed at increasing bank liquidity buffers as well as for the management of the institutions at which these policies target. It is widely accepted that liquidity creation and risk transformation are two important functions of banks in the economy, and they are somehow related. Although banks have privileged access to central bank liquidity and government-guaranteed deposits which makes them superior liquidity risk managers, when creating liquidity in the market, banks’ own liquidity is reduced and they become vulnerable to liquidity risk (Berger and Bouwman 2009). Our results suggest that the implementation of the new liquidity risk ratios (e.g. LCR and NSFR) that discourage banks from over-relying on wholesale funding would be beneficial to the economy, as reducing banks’ reliance on short-term wholesale funding increases their capacity to create liquidity which is essential for supporting the real economy with credit. The recent financial turmoil caused by COVID-19 further highlights the importance of bank liquidity creation. Banks in countries with a smaller deposit base that rely more on external financing are more likely to experience increases in the cost of funding during this period of stress (Mery and Damak 2020).

Finally, we also document that poor asset quality can positively moderate the negative relationship between wholesale funding and liquidity creation. This shows that as asset risk increases, banks are less willing to adjust their liquidity risk exposures as a response to greater reliance on wholesale funding, thus shifting the suboptimal risk to their creditors. Regulators may want to monitor moral hazard problems as well as design policy strategies to enhance bank asset quality.

**Tables and Figures**.

## Data Availability

Bank-level data is obtained from the S&P Global Market Intelligence database, while macroeconomic data is obtained from the International Monetary Fund. The University of Sheffield subscribes to the S&P Global Market Intelligence database, while International Monetary Fund data is available for free online.

## Appendix A. Liquidity classification of bank assets and variable definitions

**Table A1:** Liquidity Classification of Bank Assets

Illiquid Assets (weight = 1/2)	Semi-Liquid Assets (weight = 0)	Liquid Assets (weight = -1/2)
Corporate Loans (Loans with Maturity > 1 year)	Mortgage and Retail Loans (Loans with Maturity < = 1 year)	Cash and Cash Balances
Fixed Assets		Total Securities
Intangible Assets		Trading Assets
Other Assets		

This table illustrates the liquidity classification of bank balance sheet items and the weight assigned to each item

**Table A2:** Variable Description

Variable	Definition		Calculation		
ALC1	Asset-Side Liquidity Creation 1		(0.5*Corporate loans + 0.5*Fixed assets + 0.5*Intangible assets + 0.5*Other assets − 0.5*Cash and cash balances – 0.5*Total securities – 0.5*Trading assets)/ Total assets		
ALC2	Asset-Side Liquidity Creation 2		(0.5*Long-term loans + 0.5*Fixed assets + 0.5*Intangible assets + 0.5*Other assets − 0.5*Cash and cash balances – 0.5*Total securities – 0.5*Trading assets)/ Total assets		
ALC1-B	Asset-Side Liquidity Creation 1-B		(0.5*Corporate loans − 0.5*Cash and cash balances – 0.5*Total securities – 0.5*Trading assets)/ Total assets		
ALC2-B	Asset-Side Liquidity Creation 2-B		(0.5*Long-term loans − 0.5*Cash and cash balances – 0.5*Total securities – 0.5*Trading assets)/ Total assets		
ALC1-OBS	Asset-Side Liquidity Creation 1-Off-balance-sheet		(0.5*Credit commitments + 0.5*Corporate loans + 0.5*Fixed assets + 0.5*Intangible assets + 0.5*Other assets − 0.5*Cash and cash balances – 0.5*Total securities – 0.5*Trading assets)/ Total assets		
ALC2-OBS	Asset-Side Liquidity Creation 2-Off-balance-sheet		(0.5*Credit commitments + 0.5*Long-term loans + 0.5*Fixed assets + 0.5*Intangible assets + 0.5*Other assets − 0.5*Cash and cash balances – 0.5*Total securities – 0.5*Trading assets)/ Total assets		
CLOANS	Corporate Loans		Corporate Loans / Total Assets		
LTLOANS	Long-Term Loans		Loans with maturity > 12 months / Total Assets		
WFST	Short-Term Wholesale Funding	Financial liabilities and repurchase agreements, excluding derivatives and customer deposits with maturity < 12 months / Total Assets			
WFLT	Long-Term Wholesale Funding	Financial liabilities and repurchase agreements, excluding derivatives and customer deposits with maturity > = 12 months / Total Assets			
LLR	Loan Loss Reserves	Total loan loss and allocated transfer risk reserves / total loans and leases, net of unearned income and gross of reserve			
PL	Problem Loans	The best available in the following order: 1) Non-Performing Loans, 2) Gross Impaired Loans, 3) Net Impaired Loans, 4) Other Problem Loans (of unknown categorization) / Net Total Loans			
ROAA	Return on Average Assets	Net Income/Average Assets			
LNTA	Bank Size	Natural logarithm of total assets			
LNZSCORE	Bank Risk	Natural logarithm of the ZSCORE calculated as the sum of EQRAT and ROAA divided by the standard deviation of ROAA			
EQRAT	Equity Ratio	Total Equity/ Total Assets			
MQ	Managerial Quality	Operating Expenses/ Operating Income			
NIM	Net Interest Margin	Net Interest Income/ Average Earning Assets			
GDP	Real GDP Growth	Annual percentage change of real GDP of the host country			
UNEMP	Unemployment	The number of unemployed people as a percentage of the total labour force of the host country			
INF	Inflation	Annual percentage change of the average consumer price index (CPI) of the host country			

## Footnotes

1 The Berger and Bouwman (2009) method assigns different weights to all balance sheet items according to whether they create liquidity in the economy or withdraw funds from the market.

2 A fragile deposit structure is one that has greater reliance on the deposit base than capital.

3 Several studies document that credit scoring models increase borrower default risk (e.g. Berger et al. 2005; DeYoung et al. 2008).

4 In several cases of retail depositor runs (e.g. the collapse of Northern Rock), the runs took place after banks had already liquidated a lot of their assets to pay off their debt to short-term wholesale financiers (Goldsmith-Pinkham and Yorulmazer 2010).

5 Banks relying heavily on wholesale funding, therefore, would be forced to improve borrower monitoring to avoid withdrawals by wholesale financiers. It is well documented both theoretically (e.g. Diamond and Rajan 2000, 2001) and empirically (e.g. Berger and Bouwman 2009; Horváth et al. 2014) that financial fragility helps banks to create liquidity as monitoring is enhanced under the threat of liquidation, especially for small banks. ‘A fragile capital structure encourages the bank to commit to monitoring its borrowers, and hence allows it to extend loans. Additional equity capital makes it harder for the less-fragile bank to commit to monitoring, which in turn hampers the bank’s ability to create liquidity’ (Berger and Bouwman 2009, p. 3782).

6 We do not include the liability-side and equity components of liquidity creation because they could be mechanically affected by the use of wholesale funding by banks.

7 Our sample has relatively fewer observations for 2020.

8 Our analysis cannot eliminate the possibility that the negative relationship between wholesale funding and liquidity creation is driven by a simultaneous negative shock in the supply of deposits and credit. However, based on Fig. 1, we do not observe such system-wide shocks, suggesting that our interpretation of the results based on the liquidity exposures is more likely to hold.

9 Since most banks in our sample are non-listed, we are limited to using accounting-based measures of asset risk such as PL and LLR.

10 Researchers often use lagged variables to mitigate endogeneity concerns (e.g. Berger and Bouwman 2009; Demirgüç-Kunt and Huizinga 2010).

11 The results hold when using the average of the first three lags of our independent variables too but we avoid this option to prevent shrinkage of our sample size.

12 The two-step System GMM estimator is developed by Arellano and Bover (1995) and Blundell and Bond (1998) who augment the Arellano and Bond (1991) estimator. We use the xtabond2 Stata command by Roodman (2009) to run our regressions.

13 While the LCR and NSFR minimum requirements started being formally applied in 2015 and 2018, banks were adjusting their liquidity exposures in this direction from 2010 to prepare for the actual implementation of the ratios, which is demonstrated in Fig. 1. Therefore, we do not change the period of the sample in this test.
